# Supplementary material for: MetaRibo-Seq measures translation in microbiomes
Source: Nat Commun. 2020 Jun 29;11:3268. doi: 10.1038/s41467-020-17081-z (PMC7324362; doi:10.1038/s41467-020-17081-z)
Supplement: Supplementary file 10 — Supplementary Data 7 [file 41467_2020_17081_MOESM10_ESM.zip › File2/Confidence_High_Taxonomy/57011_out.krona.html]

Javascript must be enabled to view this page.

members
magnitude
magnitudeUnassigned
count
unassigned
taxon
rank

57011\_out

279

279
2
superkingdom

976
278
phylum

class
278
200643

278

SRS012849\_contig\_number\_contig-100\_19380.19380SRS019381\_contig\_number\_8178SRS144135\_contig\_number\_contig-100\_6100.177153SRS146812\_contig\_number\_contig-100\_15498.234375
171549
order
4

family
29
815

genus
2
816
29

SRS143523\_contig\_number\_20761SRS893383\_contig\_number\_26505


SRS021948\_contig\_number\_21555
1
204516
species

species
47678
4

SRS017701\_contig\_number\_10166SRS077589\_contig\_number\_805SRS101376\_contig\_number\_790SRS971276\_contig\_number\_109

species
821
3

SRS050299\_contig\_number\_contig-100\_3715.34189SRS064557\_contig\_number\_3146SRS104693\_contig\_number\_10619

species

SRS045645\_contig\_number\_24435
1
1262734

1
1638779

SRS019910\_contig\_number\_contig-100\_136.131537
species

1

SRS077392\_contig\_number\_27657
671267
species

species
2
817

SRS017521\_contig\_number\_45433SRS074964\_contig\_number\_454

1
310298

SRS014459\_contig\_number\_30903
species

1
818

SRS052027\_contig\_number\_11453
species

species

SRS011084\_contig\_number\_5262SRS016267\_contig\_number\_10319
2
626929

species

SRS064645\_contig\_number\_1968
1
2292944

1
338188

SRS063040\_contig\_number\_23490
species

1

SRS053649\_contig\_number\_335
1262737
species

species
1739517
1

SRS098717\_contig\_number\_5780

species
291644
1

SRS013800\_contig\_number\_6866

species

SRS893253\_contig\_number\_490
1
28116


SRS012273\_contig\_number\_31818SRS046369\_contig\_number\_13777SRS077454\_contig\_number\_11181SRS148511\_contig\_number\_2415
4
46506
species

1
171552
family

genus
838
1

species
1
59823

SRS097920\_contig\_number\_26594

236
2005525
family

95
genus
236
375288

SRS011134\_contig\_number\_10859SRS012969\_contig\_number\_29177SRS014412\_contig\_number\_5635SRS014683\_contig\_number\_9005SRS014948\_contig\_number\_contig-100\_1690.137982SRS015369\_contig\_number\_1627SRS015431\_contig\_number\_57629SRS016056\_contig\_number\_2446SRS016335\_contig\_number\_23388SRS016495\_contig\_number\_10322SRS016629\_contig\_number\_3891SRS016954\_contig\_number\_12431SRS017433\_contig\_number\_2745SRS018351\_contig\_number\_12255SRS019030\_contig\_number\_11987SRS019068\_contig\_number\_74203SRS019808\_contig\_number\_contig-100\_2591.124272SRS020622\_contig\_number\_4284SRS021219\_contig\_number\_4817SRS022609\_contig\_number\_2037SRS023526\_contig\_number\_27576SRS023715\_contig\_number\_contig-100\_1842.163423SRS024331\_contig\_number\_27361SRS024625\_contig\_number\_contig-100\_1083.104906SRS043667\_contig\_number\_contig-100\_287.41721SRS043768\_contig\_number\_28095SRS047014\_contig\_number\_30491SRS048060\_contig\_number\_1665SRS048164\_contig\_number\_25431SRS048981\_contig\_number\_4038SRS049402\_contig\_number\_7987SRS049773\_contig\_number\_17674SRS049959\_contig\_number\_4564SRS050026\_contig\_number\_9861SRS050422\_contig\_number\_33760SRS050974\_contig\_number\_1569SRS052697\_contig\_number\_29355SRS053087\_contig\_number\_contig-100\_5096.42923SRS053573\_contig\_number\_1899SRS056519\_contig\_number\_4229SRS056537\_contig\_number\_18641SRS058723\_contig\_number\_8356SRS058770\_contig\_number\_14841SRS063370\_contig\_number\_12430SRS064757\_contig\_number\_8395SRS065176\_contig\_number\_5890SRS065504\_contig\_number\_2818SRS075021\_contig\_number\_8319SRS075773\_contig\_number\_28181SRS075821\_contig\_number\_contig-100\_5001.117644SRS075878\_contig\_number\_17898SRS076976\_contig\_number\_17844SRS077231\_contig\_number\_contig-100\_4969.149116SRS077641\_contig\_number\_contig-100\_3981.125212SRS077730\_contig\_number\_9880SRS077849\_contig\_number\_20746SRS078242\_contig\_number\_23120SRS078665\_contig\_number\_6892SRS098571\_contig\_number\_44427SRS098655\_contig\_number\_12175SRS101268\_contig\_number\_contig-100\_2155.16074SRS1041038\_contig\_number\_12524SRS1041039\_contig\_number\_26053SRS1041136\_contig\_number\_15817SRS1041137\_contig\_number\_22638SRS1041140\_contig\_number\_5497SRS1041144\_contig\_number\_2544SRS1041147\_contig\_number\_1886SRS1054716\_contig\_number\_contig-100\_3355.82075SRS1055043\_contig\_number\_contig-100\_3444.153693SRS1055056\_contig\_number\_contig-100\_4147.86987SRS1055069\_contig\_number\_13506SRS1055076\_contig\_number\_14570SRS1055099\_contig\_number\_6501SRS140492\_contig\_number\_6557SRS142503\_contig\_number\_39449SRS142599\_contig\_number\_24102SRS143070\_contig\_number\_contig-100\_23497.23497SRS143085\_contig\_number\_3421SRS143722\_contig\_number\_14613SRS144183\_contig\_number\_25088SRS147039\_contig\_number\_3342SRS147653\_contig\_number\_2545SRS148721\_contig\_number\_33719SRS148784\_contig\_number\_26577SRS148970\_contig\_number\_contig-100\_2956.38742SRS149075\_contig\_number\_16564SRS149181\_contig\_number\_23037SRS150029\_contig\_number\_16049SRS893172\_contig\_number\_12789SRS893187\_contig\_number\_11733SRS893230\_contig\_number\_12457SRS893270\_contig\_number\_contig-100\_3688.126008SRS893341\_contig\_number\_15846SRS893380\_contig\_number\_contig-100\_1973.33236


SRS013951\_contig\_number\_31779SRS019161\_contig\_number\_31761SRS1041133\_contig\_number\_26728
3
2293116
species

species

SRS011239\_contig\_number\_14956SRS013098\_contig\_number\_34716SRS013158\_contig\_number\_4233SRS013638\_contig\_number\_5757SRS014235\_contig\_number\_46150SRS014313\_contig\_number\_4162SRS015065\_contig\_number\_28095SRS015579\_contig\_number\_21368SRS015663\_contig\_number\_17127SRS015794\_contig\_number\_9852SRS015890\_contig\_number\_11590SRS016095\_contig\_number\_20905SRS016989\_contig\_number\_12599SRS017191\_contig\_number\_16588SRS017307\_contig\_number\_27314SRS018313\_contig\_number\_10046SRS018541\_contig\_number\_13029SRS018656\_contig\_number\_12238SRS018888\_contig\_number\_2177SRS018984\_contig\_number\_9295SRS019178\_contig\_number\_2812SRS019267\_contig\_number\_6495SRS019601\_contig\_number\_25204SRS019685\_contig\_number\_40611SRS019787\_contig\_number\_9983SRS020394\_contig\_number\_4715SRS022713\_contig\_number\_5031SRS023583\_contig\_number\_2335SRS023829\_contig\_number\_168SRS023971\_contig\_number\_708SRS024132\_contig\_number\_19689SRS024388\_contig\_number\_9065SRS024435\_contig\_number\_31952SRS043701\_contig\_number\_14414SRS044535\_contig\_number\_13197SRS045195\_contig\_number\_6292SRS045713\_contig\_number\_10626SRS045826\_contig\_number\_13021SRS046712\_contig\_number\_607SRS049712\_contig\_number\_19984SRS049900\_contig\_number\_11329SRS050752\_contig\_number\_contig-100\_335.169205SRS051031\_contig\_number\_34461SRS051610\_contig\_number\_8637SRS051882\_contig\_number\_10092SRS052078\_contig\_number\_435SRS053398\_contig\_number\_16964SRS056259\_contig\_number\_19154SRS056273\_contig\_number\_24444SRS058145\_contig\_number\_contig-100\_25.34628SRS062701\_contig\_number\_5794SRS065397\_contig\_number\_13383SRS075398\_contig\_number\_2756SRS075984\_contig\_number\_19826SRS076804\_contig\_number\_5120SRS077127\_contig\_number\_14947SRS077194\_contig\_number\_21777SRS077335\_contig\_number\_7606SRS078176\_contig\_number\_20225SRS078419\_contig\_number\_1017SRS097958\_contig\_number\_13786SRS098061\_contig\_number\_2740SRS100021\_contig\_number\_7781SRS103987\_contig\_number\_25508SRS104036\_contig\_number\_8217SRS1041032\_contig\_number\_12332SRS1041036\_contig\_number\_824SRS1041090\_contig\_number\_2298SRS1041091\_contig\_number\_18391SRS1041095\_contig\_number\_2379SRS1041132\_contig\_number\_1873SRS1041134\_contig\_number\_4604SRS1041138\_contig\_number\_13209SRS1041142\_contig\_number\_3625SRS1041145\_contig\_number\_6408SRS104311\_contig\_number\_19972SRS104327\_contig\_number\_7735SRS1055038\_contig\_number\_2815SRS1055049\_contig\_number\_11892SRS142542\_contig\_number\_contig-100\_333.162203SRS142712\_contig\_number\_1SRS142890\_contig\_number\_37253SRS143148\_contig\_number\_15767SRS143342\_contig\_number\_17363SRS143876\_contig\_number\_24163SRS144297\_contig\_number\_contig-100\_1837.99339SRS144362\_contig\_number\_36241SRS144506\_contig\_number\_5944SRS144603\_contig\_number\_11145SRS144714\_contig\_number\_14026SRS145497\_contig\_number\_13942SRS146764\_contig\_number\_16265SRS146813\_contig\_number\_26511SRS147272\_contig\_number\_925SRS147377\_contig\_number\_9798SRS147614\_contig\_number\_7700SRS148196\_contig\_number\_28396SRS148253\_contig\_number\_14182SRS148817\_contig\_number\_10789SRS148874\_contig\_number\_11146SRS893256\_contig\_number\_1361SRS893259\_contig\_number\_2495SRS893285\_contig\_number\_875SRS893288\_contig\_number\_1422SRS893342\_contig\_number\_8452SRS893382\_contig\_number\_2072SRS971275\_contig\_number\_24306
107
823

species
1

SRS017103\_contig\_number\_10525
469591

species
1
387661

SRS015960\_contig\_number\_12783

46503
6

SRS018836\_contig\_number\_15100SRS045004\_contig\_number\_19488SRS045244\_contig\_number\_3154SRS1041092\_contig\_number\_8867SRS149244\_contig\_number\_8032SRS893231\_contig\_number\_1350
species

2293123
8

SRS014287\_contig\_number\_18684SRS015217\_contig\_number\_27791SRS018575\_contig\_number\_5733SRS019397\_contig\_number\_1710SRS019968\_contig\_number\_15024SRS054590\_contig\_number\_1751SRS077294\_contig\_number\_12440SRS893252\_contig\_number\_17866
species

species
1
1263091

SRS050941\_contig\_number\_18393

1262912
7

SRS019496\_contig\_number\_14659SRS020233\_contig\_number\_41581SRS047044\_contig\_number\_contig-100\_2230.95144SRS050925\_contig\_number\_25233SRS050998\_contig\_number\_14516SRS1041118\_contig\_number\_6161SRS1041129\_contig\_number\_13478
species

3
2293114

SRS053214\_contig\_number\_16236SRS057478\_contig\_number\_6019SRS064973\_contig\_number\_1157
species

species
1

SRS075078\_contig\_number\_24790
1947048

species
3
2293117

SRS018623\_contig\_number\_8461SRS020328\_contig\_number\_26436SRS022071\_contig\_number\_29026

171551
7
family

genus
836
7

7

SRS015190\_contig\_number\_11365SRS015264\_contig\_number\_15758SRS019445\_contig\_number\_28191SRS043411\_contig\_number\_contig-100\_635.107013SRS077086\_contig\_number\_11194SRS104975\_contig\_number\_15199SRS147346\_contig\_number\_52493
658663
species

1853231
1
family

283168
1
genus

species

SRS048870\_contig\_number\_20059
1
1637502

phylum
1
1239

186801
1
class

order
186802
1

family
541000
1

genus
1
1263

species
2293181
1

SRS147271\_contig\_number\_24674
